# The effects of DLEU1 gene expression in Burkitt lymphoma (BL): potential mechanism of chemoimmunotherapy resistance in BL

**DOI:** 10.18632/oncotarget.15711

**Published:** 2017-02-24

**Authors:** Sanghoon Lee, Wen Luo, Tishi Shah, Changhong Yin, Timmy O’Connell, Tae-Hoon Chung, Sherrie L Perkins, Rodney R Miles, Janet Ayello, Erin Morris, Lauren Harrison, Carmella van de Ven, Mitchell S Cairo

**Affiliations:** ^1^ Departments of Pediatrics, New York Medical College, Valhalla, New York, USA; ^2^ Departments of Cell Biology and Anatomy, New York Medical College, Valhalla, New York, USA; ^3^ Departments of Microbiology and Immunology, New York Medical College, Valhalla, New York, USA; ^4^ Cancer Science Institute of Singapore, National University of Singapore, Singapore; ^5^ Department of Pathology and ARUP Laboratories, University of Utah, Salt Lake City, Utah, USA; ^6^ Departments of Pathology, New York Medical College, Valhalla, New York, USA; ^7^ Departments of Medicine, New York Medical College, Valhalla, New York, USA

**Keywords:** DLEU1, tumor suppressor, chemoimmunotherapy, genome editing, B-NHL

## Abstract

Following a multivariant analysis we demonstrated that children and adolescents with Burkitt lymphoma (BL) and a 13q14.3 deletion have a significant decrease in event free survival (EFS) despite identical short intensive multi-agent chemotherapy. However, how this deletion in the 13q14.3 region is associated with a significant decrease in EFS in children and adolescents with BL is largely unknown. The gene Deleted in Lymphocytic Leukemia 1 (*DLEU1*) is located in the region of 13q14.3. Here, we report that DLEU1 expression is implicated in the regulation of BL programmed cell death, cell proliferation, and expression of apoptotic genes in transcription activator-like effector nuclease (TALEN)s-induced *DLEU1* knockdown and DLEU1 overexpressing BL cell lines. Furthermore, NSG mice xenografted with *DLEU1* knockdown BL cells had significantly shortened survival (*p* < 0.05 and *p* < 0.005), whereas those xenografted with DLEU1 overexpressing BL cells had significantly improved survival (*p* < 0.05 and *p* < 0.0001), following treatment with rituximab and/or cyclophosphamide. These data suggest that *DLEU1* may in part function as a tumor suppressor gene and confer chemoimmunotherapy resistance in children and adolescents with BL.

## INTRODUCTION

Pediatric Burkitt lymphoma (BL) is the most common histological subtype of non-Hodgkin lymphoma (NHL) in children and adolescents [1, 2]. We and others have demonstrated that the prognosis of pediatric BL has remarkably improved over the past 40 years following the introduction of short intensive multi-agent chemotherapy [1–4]. However, this successful approach is limited by significant chemotherapy-induced acute toxicity, suggesting the need to identify less toxic and more targeted therapy [1, 2]. We investigated the addition of the anti-CD20 monoclonal antibody, rituximab, to the FAB (French American British) chemotherapy regimen in children and adolescents with intermediate-risk and advanced BL [5, 6]. Immunotherapy, by targeting CD20 by rituximab, has been demonstrated to alter a variety of signal transduction pathways, including NF-κB, MAPK/ERK, JAK/STAT, PI3K/AKT, and B-cell receptor pathways. These alterations lead to cell death and/or sensitization of cells to the effects of cytotoxic chemotherapeutic agents [7–9]. Furthermore, children and adolescents with BL who relapse or progress after initial chemoimmunotherapy have a dismal prognosis, suggesting the development of chemoimmunotherapy resistant disease [1, 2]. Novel strategies will be required for the reduction of both acute morbidities and prolonged hospital stays secondary to intensive multi-agent chemotherapy in newly diagnosed patients with BL and/or circumvention of chemoimmunotherapy resistance in patients with relapsed/refractory disease.

We have previously identified secondary chromosomal aberrations in 70% of pediatric BL patients with a *C-MYC* gene rearrangement [10]. Specifically, we identified a significantly inferior event free and overall survival (EFS and OS) in children and adolescents with a specific loss of the 13q14.3 locus [10, 11]. In multivariate analysis controlling for stage, lactate dehydrogenase (LDH) levels, country of treatment, and group classification, children and adolescents with BL who had a 13q deletion had significantly poorer EFS compared to patients treated with the same French American British (FAB) chemotherapy regimen [10].

We compared the molecular signature and gene expression profile of pediatric BL patients treated within the Children’s Oncology Group, National Cancer Institute, and Berlin-Frankfurt-Munster (BFM) - pediatric NHL Group [12] and found consistency in the pediatric BL molecular signature [12–15]. Interestingly, Dave *et al*. suggested that *DLEU1* gene located at chromosome 13q14.3 region [16, 17] is significantly amplified in adult BL *vs*. diffuse large B-cell lymphoma (DLBCL), and is one of the BL molecular classifying genes and a target of the oncogene, *C-MYC* [14]. Deletion of *DLEU1*, therefore, may in part form the molecular basis for the significantly inferior EFS in children with BL and a concomitant 13q14.3 deletion when treated with FAB chemotherapy [10].

*DLEU1* has been found frequently deleted and a potential tumor suppressor gene in hematopoietic tumors including chronic lymphocytic leukemia (CLL) and mantle cell lymphoma [16, 17]. An open reading frame corresponding to a 78 amino acids sequence has been identified in *DLEU1* gene by human transcriptome, functional genomics and proteomic analysis [18, 19]. DLEU1 protein has been predicted to interact with several cancer-related proteins, including c-Myc, Tubulin beta-2C chain (TUBB2C), E3 ubiquitin-protein ligase (UBR1), cellular tumor antigen p53, and Ras association (RalGDS/AF-6) domain family member 1 (RASSF1) [18]. Interestingly, TUBB2C and RASSF1 are frequently silenced in human cancers and enhance apoptosis and tumor suppression [20, 21]. UBR1 affects the cell cycle via PI3K-AKT signaling and loss of UBR1 accelerates B-cell lymphomagenesis [22]. We have observed that the expression levels of RASSF1, TUBB2C and UBR1 were significantly higher in BL compared to DLBCL cell lines [23]. These data, taken together, suggest that DLEU1 may function as a tumor suppressor in c-Myc activated BL by repressing cell cycle progression and enhancing programmed cell death via protein-protein interaction. In the current study, we set out to investigate the hypothesis that the deletion of *DLEU1* in BL may affect the expression of *DLEU1* network genes and alter signal transduction pathways leading to the inhibition of programmed cell death and in part be responsible for the mechanism of resistance to chemoimmunotherapy in patients with BL with a 13q14.3 deletion.

## RESULTS

### Generation of TALEN mediated DLEU1 knockdown BL cell line

**Figure 1:**
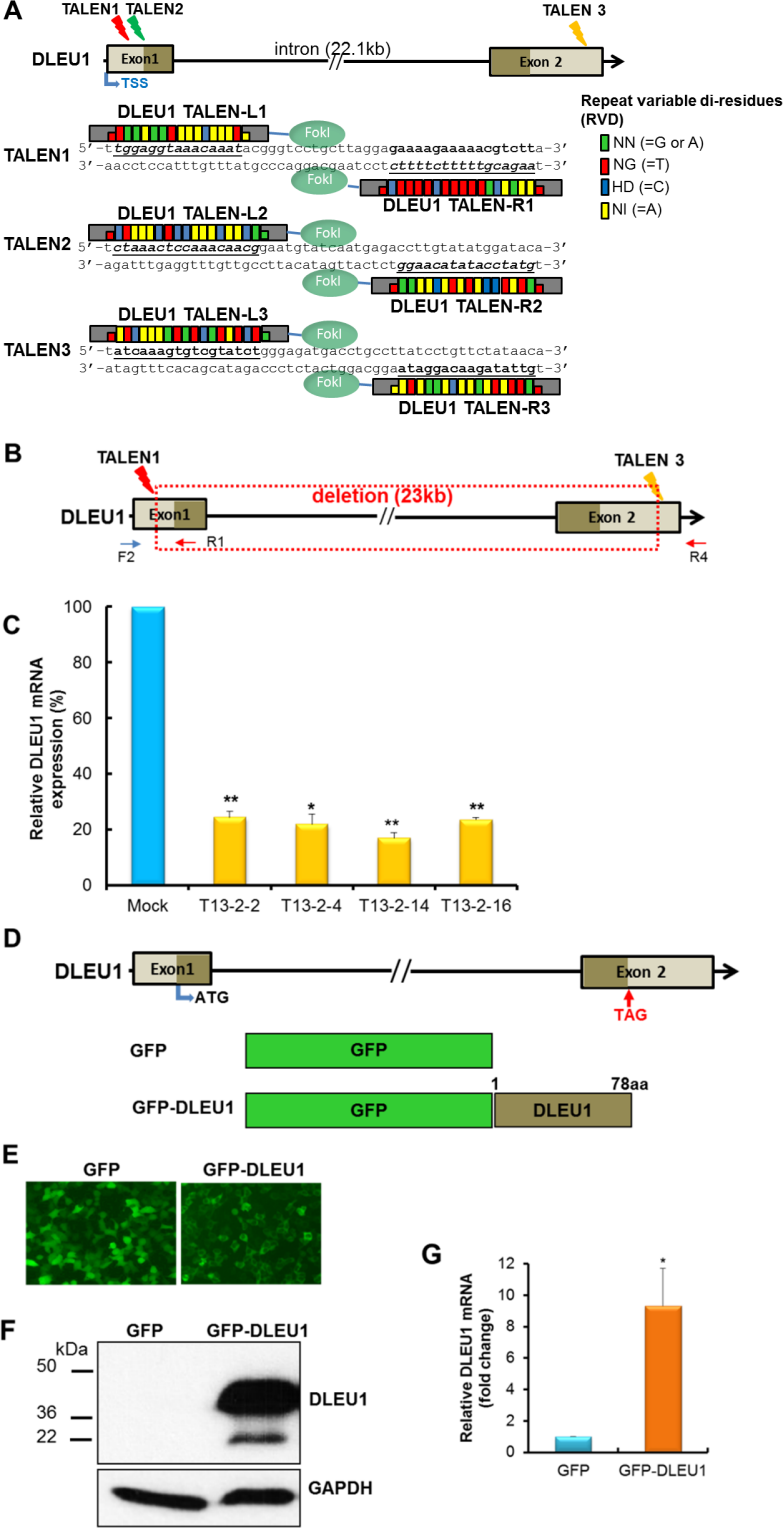
TALENs-induced *DLEU1* knockdown and DLEU1 stably overexpressing Raji cell line (**A**) A diagram showing the targeting of the DLEU1 locus by three different TALENs and TALE repeat arrays are shown with the repeat-variable di-residue (RVD) underlined and represented by small colored rectangles boxes. Two monomeric TALENs (L and R) are required to bind the *DLEU1* target site to cleave DNA by fused *Fok*I. TSS, transcription start site. (**B**) Single clones with TALEN1 and 3-induced *DLEU1* locus disruption (22,843bp deletion, boxed with red dot line) were isolated. (**C**) Comparison of expression of DLEU1 mRNA between WT control (WT) and DLEU1 knockdown single clones T13-2-2, T13-2-4, T13-2-14 and T13-2-16 by qRT-PCR. Data are presented as mean ± SD (*n* = 3, paired *t* test) and *p*-value are displayed on plot. (**D**) Diagram of DLEU1 gene and ORF (78 a.a). Empty GFP vector (GFP) and GFP fused DLEU1 (GFP-DLEU1) constructs are shown. ATG, start codon; TAG, stop codon. (**E**) GFP and GFP-DLEU1 fusion plasmids were stably transfected into Raji cells, and then western blotting (**F**) and qRT-PCR (**G**) were performed. Data are presented mean ± SD of triplicates (paired *t* test). *p < 0.005.

Three pairs of TALENs (TALEN1, TALEN2, and TALEN3) targeting the endogenous *DLEU1* gene were constructed based on modified restriction enzyme and ligation (REAL) assembly methods for *DLEU1* gene modification (Figure 1A). To excise the entire *DLEU1* locus, TALEN1 and TALEN3 (T13), and TALEN2 and TALEN3 (T23) were transfected into Raji cells. Single *DLEU1* knockdown Raji cell clones were screened for the desired 23 Kb deletion which was confirmed by PCR and sequencing analysis (Figure 1B). To ensure the purity of a single clone, one of the positive single clones (T13-2) was re-plated and its daughter cells, four clones T13-2-2, T13-2-4, T13-2-14 and T13-2-16 were re-screened as above. Quantitative RT-PCR showed significant reduction in expression of *DLEU1* compared to WT, with reductions of 75% (*p* < 0.01), 80% (*p* < 0.05), 83% (*p* < 0.01), and 77% (*p* < 0.01) in clones T13-2-2, T13-2-4, T13-2-14, and T13-2-16, respectively (Figure 1C). Since clone T13-2-14, hereafter referred to as “*DLEU1* knockdown (DLEU1-KD)”, showed the highest reduction of *DLEU1* mRNA, we used this clone for all further experimentation in this study. The DLEU1-KD clones had no significant reduction in DLEU2 mRNA (data not shown).

### Establishment of DLEU1 stably overexpressing BL cell line

DLEU1 full-length cDNA was cloned into pEGFP-N3 expression vector and GFP-DLEU1 plasmid was transfected into HEK293 cells to confirm expression of the fusion protein under the fluorescent microscope (Figure 1D and 1E). GFP-DLEU1 construct was then stably transfected into Raji cells. The expression of DLEU1 at mRNA level was detected by RT-PCR (Figure 1F), and the predicted size of the fusion protein (approximately 36 kDa) was confirmed by western blotting analysis (Figure 1G) whereas endogenous DLEU1 protein was not detectable in GFP control.

### DLEU1 expression levels have significant effects on BL cell proliferation and programmed cell death

**Figure 2:**
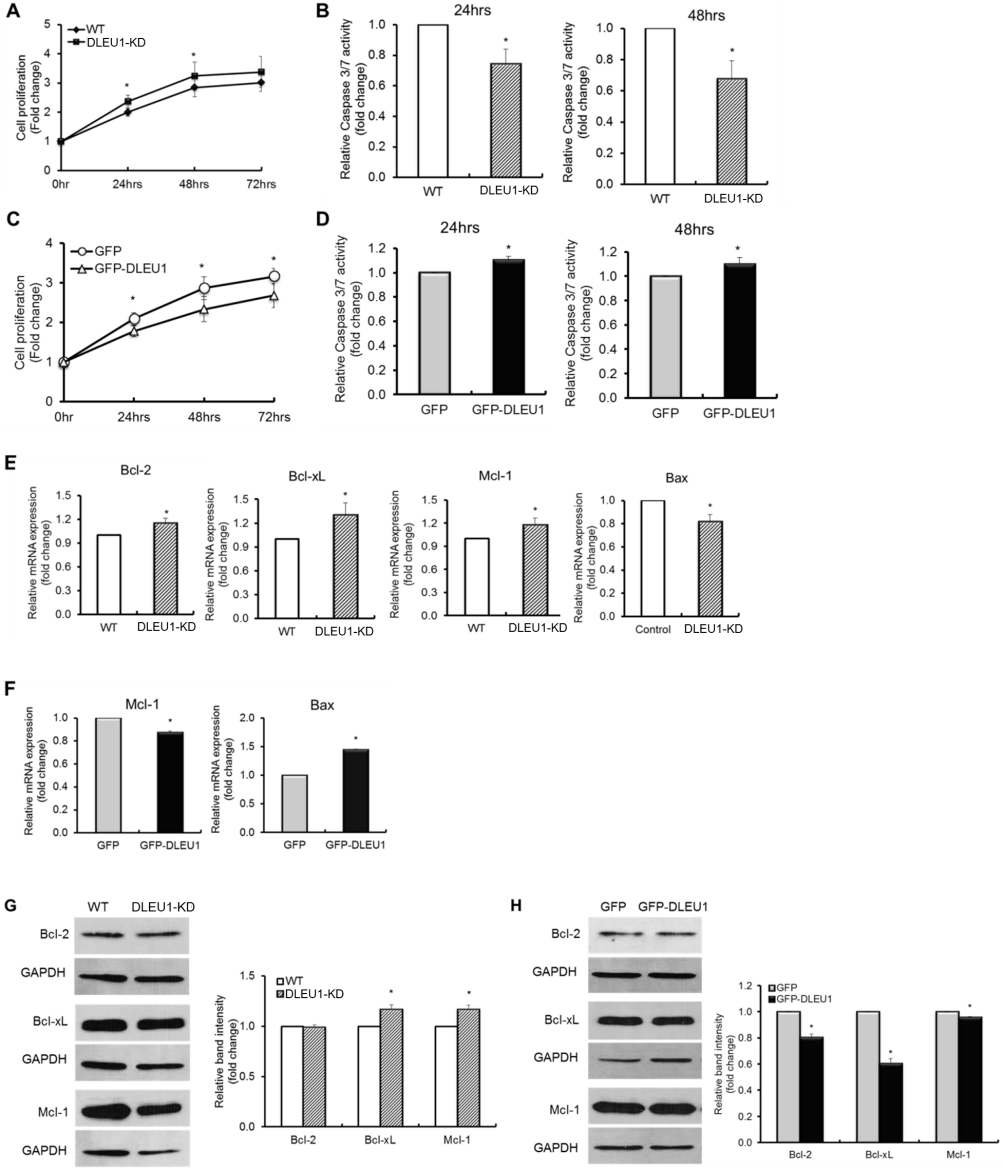
DLEU1 downregulates cell proliferation and upregulates caspase-dependent apoptosis (**A**, **C**) Comparison of DLEU1-KD vs WT (A) and GFP-DLEU1 vs GFP (C) cell proliferation. (**B**, **D**) Caspase 3/7 activity in DLEU1-KD vs WT cells (B) and GFP-DLEU1 vs GFP cells (D). (**E**–**H**) mRNA (E, F) and protein (G, H) expression of anti-apoptotic and pro-apoptotic genes in the Bcl-2 family in DLEU1-KD vs WT (E, G) and GFP-DLEU1 vs GFP (F, H) cells. Data are represented as the mean ± SD of triplicates (paired *t* test). *p < 0.05; **p < 0.01.

To examine whether *DLEU1* gene affects cell proliferation and programmed cell death in BL, Raji cells with or without DLEU1 knocked out (DLEU1-KD or WT), were plated into 48-well plates and evaluated every 24 hours for cell proliferation by MTS assay and programmed cell death by Caspase 3/7 assay. DLEU1-KD cells showed a significant increase in cell proliferation (20% at 24 hrs, *p* < 0.05; 25% at 48 hrs, *p* < 0.05) (Figure 2A), and a significant reduction in caspase 3/7 activity (25% at 24 hrs, *p* < 0.01; 33% at 48 hrs, *p* < 0.05) (Figure 2B), when compared to those of WT cells. Conversely, when we overexpressed GFP or GFP-DLEU1 in Raji cells, GFP-DLEU1 cells showed a significant decrease in cell proliferation (15% reduction, *p* < 0.03; 19% reduction, *p* < 0.03; and 15% reduction, *p* < 0.01 at 24, 48 and 72 hours, respectively) (Figure 2C) and a significant increase in programmed cell death (10.2% increase, *p* < 0.02; 9.7% increase, *p* < 0.05 at 24 and 48 hours, respectively) (Figure 2D) compared to GFP control cells. We further analyzed the expression of anti- and pro-apoptotic genes in these cells, and found that DLEU1 knockdown resulted in a significant increase in expression of the anti-apoptotic genes (*Bcl-2*, *Bcl-xL* and *Mcl-1*), and decrease in that of the pro-apoptotic gene (*Bax)* at mRNA level (Figure 2E). Consistently, GFP-DLEU1 expression induced a significant decrease in *Mcl-1* and increase in *Bax* expression (Figure 2F). We observed similar changes in the expression of these genes at protein levels (Figure 2G and 2H).

### DLEU1 significantly affects the expression of network genes and signaling pathways in BL

**Figure 3:**
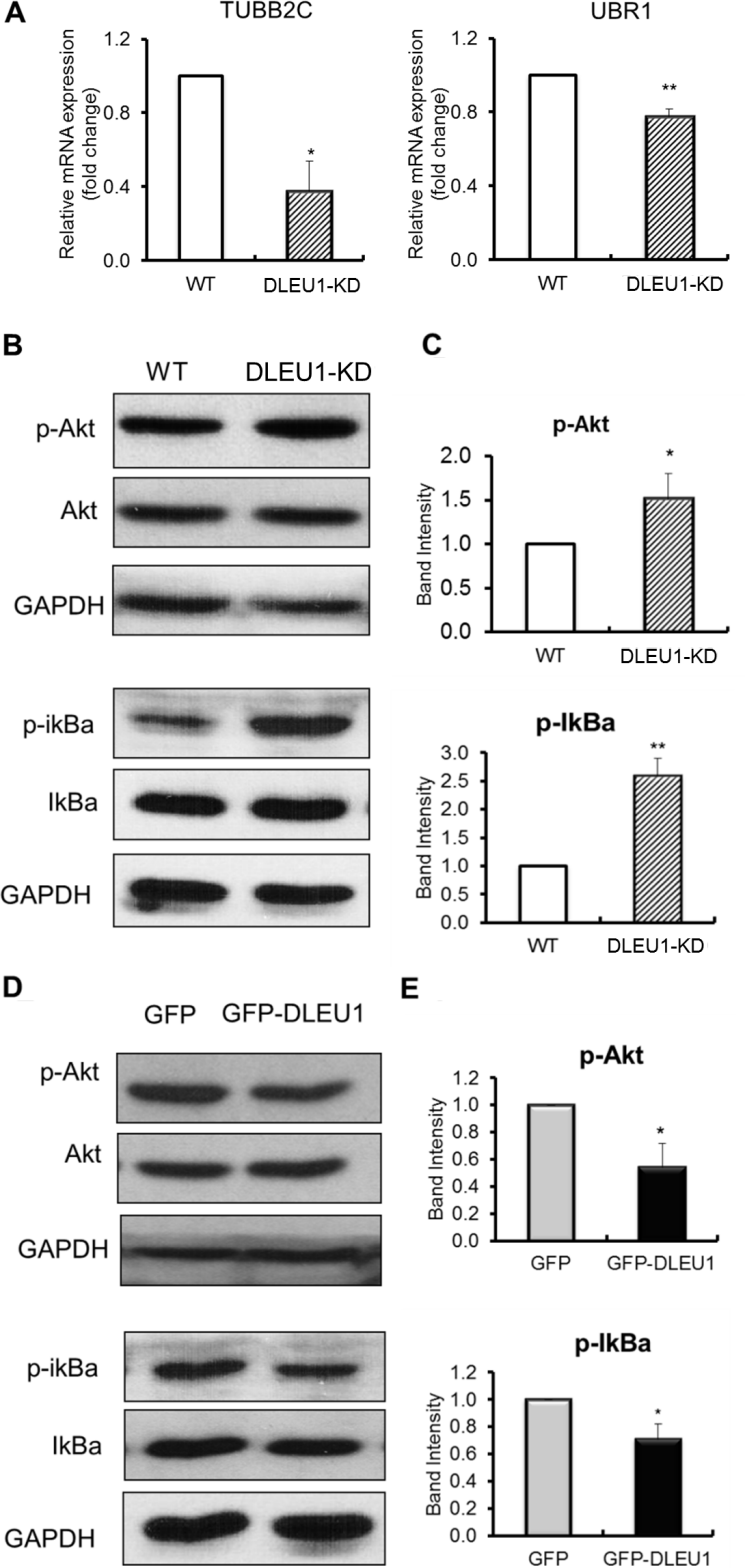
DLEU1 regulates the expression of network genes and signaling pathways (**A**) mRNA expression of *DLEU1* network genes in DLEU1-KD vs WT cells. (**B**, **C**) Significant increase in p-Akt and p-IκBα levels in DLEU1-KD vs WT cells. (**D**, **E**) Significant decrease in p-Akt and p-IκBα levels in GFP-DLEU1 vs GFP cells. Data are represented as the mean ± SD of triplicates (paired *t* test). *p < 0.05; **p < 0.01.

To monitor the expression of *DLEU1* network genes, *TUBB2C* and *UBR1*, in DLEU1-KD cells compared to WT, quantitative reverse-transcriptase PCR (qRT-PCR) was performed. There was significantly reduced expression of *TUBB2C* and *UBR1* mRNA (60%, *p* < 0.05; 25%, *p* < 0.01, respectively) (Figure 3A) in DLEU1-KD compared to WT cells. Furthermore, we compared protein activation related to Akt and NF-κB signaling pathways in DLEU1-KD and WT cells. DLEU1 knockdown resulted in significant increase in phosphorylation level of IκBα (2.6 fold, *p* < 0.01) and Akt (1.5 fold, *p* < 0.05) (Figure 3B and 3C). Conversely, GFP-DLEU1 expression induced significant inhibition of phosphorylation of IκBα (30% reduction, *p* < 0.05) and Akt (46% reduction, *p* < 0.05) (Figure 3D and 3E).

### DLEU1 knockdown results in significant inhibition of programmed cell death, and increase in cell proliferation, in rituximab and/or cyclophosphamide treated cells

**Figure 4:**
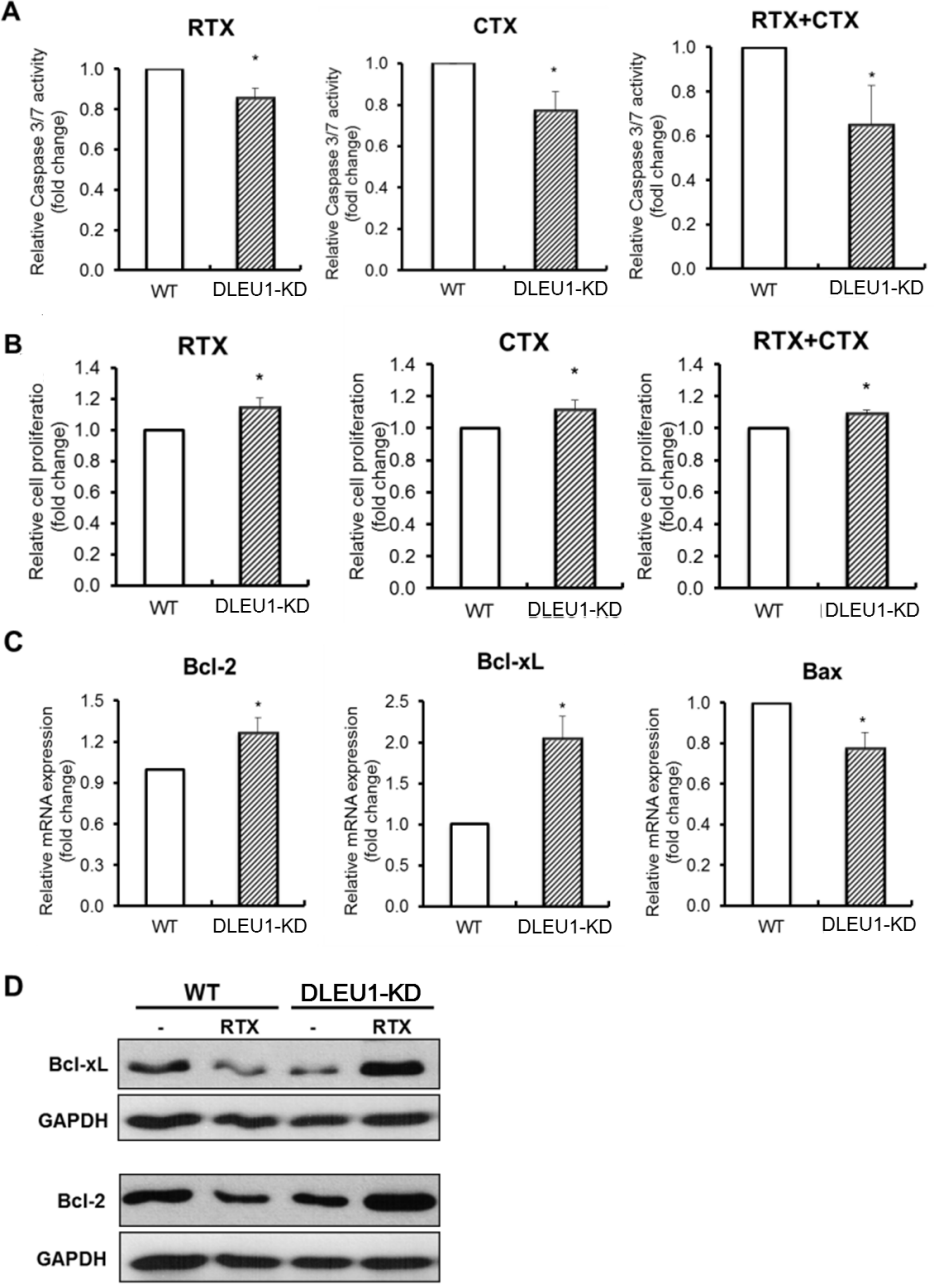
Caspase-dependent apoptosis and cell proliferation in rituximab and cyclophosphamide alone or in combination-treated DLEU1-KD vs WT cells (**A**) Caspase 3/7 activity of rituximab (RTX, 10 ug/ml), cyclophosphamide (CTX, 10 mM) and RTX plus CTX combination (RTX/CTX) treated DLEU1-KD vs WT cells. (**B**) Cell proliferation of RTX, CTX and RTX/CTX treated DLEU1-KD vs WT cells. (**C**, **D**) The mRNA (C) and protein (D) expression of anti-apoptotic genes in RTX treated DLEU1-KD vs WT cells. Data are represented as the mean ± SD of triplicates (paired *t* test). *p < 0.05.

We next examined the changes in cell proliferation and caspase 3/7 dependent apoptosis in DLEU1-KD cells. These cells were treated with rituximab (RTX) (10 ug/ml) and cyclophosphamide (CTX) (10 mM), alone or in combination, for 48 hours. A significant reduction was noted in caspase 3/7-dependent apoptosis in DLEU1-KD cells treated with RTX alone (15% reduction, *p* < 0.05), CTX alone (24% reduction, *p* < 0.05) and in combination (35% reduction, *p* < 0.05) compared to WT cells (Figure 4A). Conversely, a significant increase in cell proliferation was observed in DLEU1-KD cells treated with RTX alone (10% increase, *p* < 0.05), CTX alone (9.4% increase, *p* < 0.05) and in combination (7.7% increase, *p* < 0.05) (Figure 4B). Moreover, RTX treated DLEU1-KD cells showed a significant increase in expression of anti-apoptotic genes, *Bcl-2* and *Bcl-xL* (>1.3-fold, *p* < 0.05 and >2.0-fold, *p* < 0.05, respectively), and a significant decrease in expression of the pro-apoptotic gene, *Bax* (~25%, *p* < 0.05) (Figure 4C). These results were further confirmed at protein level by western blotting analyses. (Figure 4D).

### Global gene expression profiling in DLEU1 knockdown cells

**Figure 5:**
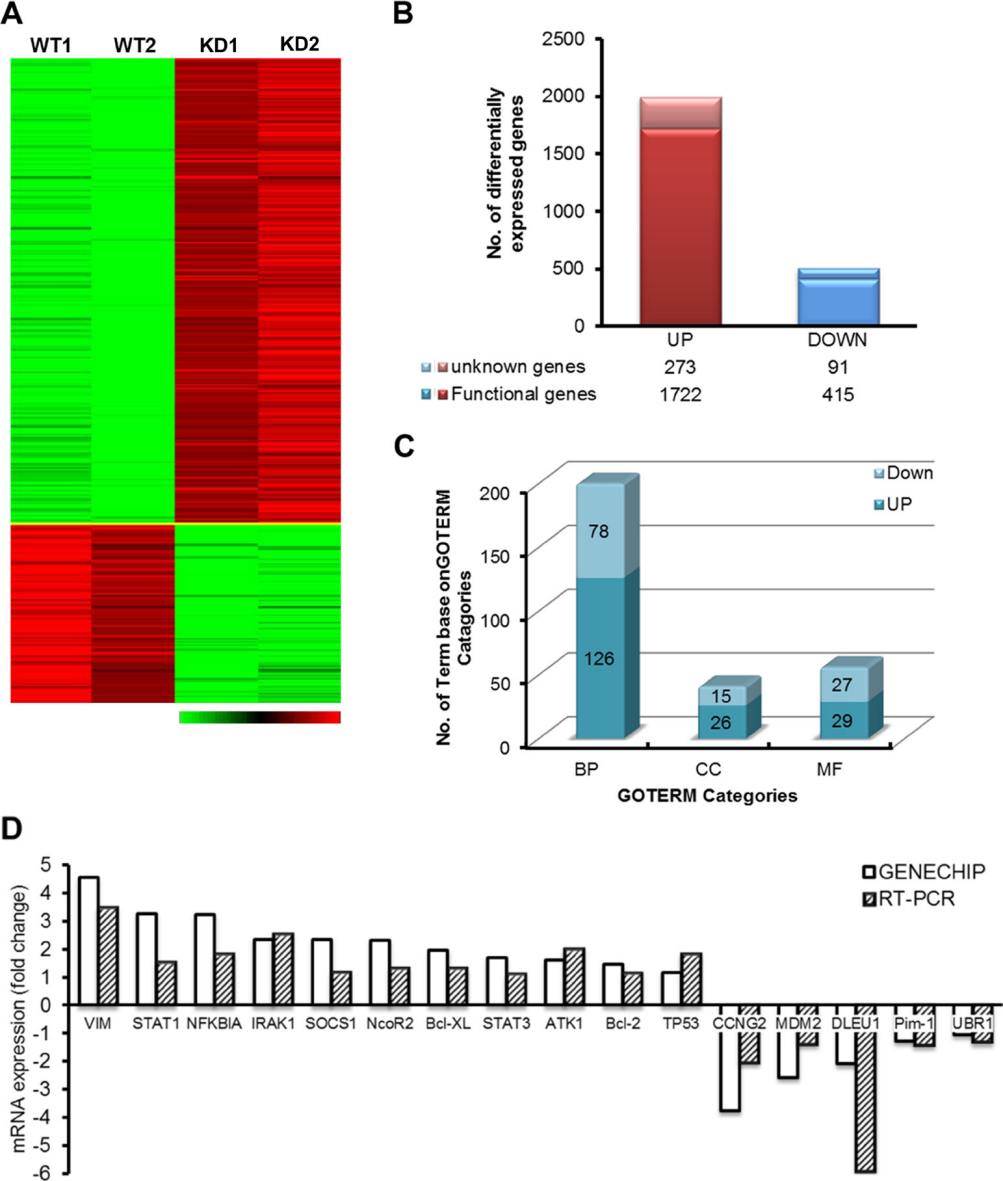
Gene expression patterns in DLEU1-KD *vs* WT (**A**) Hierarchical clustering comparing the gene expression patterns between DLEU1-KD and WT cells. Green represents decreased expression and red increased expression, according to the scale. (**B**) Number of up- (left column) and down- (right column) regulated genes by DLEU1. (**C**) The numbers of genes in each functional category are demonstrated. (**D**) Verification of DLEU1 regulated genes identified by Affymetrix Genechip profiling by qRT-PCR. GAPDH was used as an endogenous control for normalization.

To understand and elucidate the function and mechanism of *DLEU1* gene in BL, we took a genomic approach employing Affymetrix GeneChip technology in DLEU1-KD vs. WT cells. A total of 2,501 differentially expressed genes (DEGs) from 21,381 human genes were identified (>2-fold, *p* < 0.05, 11.7%) between TALENs-induced DLEU1-KD cells compared to WT cells (Figure 5A). From among the 2,501 filtered genes, 1,995 genes (1,722 known genes plus 273 unknown genes, 79.8%) were up-regulated and 506 genes (415 known genes plus 91 unknown genes, 20.2%) were down-regulated (Figure 5B). When DEG-associated gene ontology (GO) terms were assessed, 126, 26 and 29 GO terms were associated with up-regulated genes whereas 78, 15 and 27 GO terms were associated with down-regulated genes (*p* < 0.05) among biological process, cellular component, and molecular function categories, respectively (Figure 5C and Supplementary Table 1). Functional clustering results of up-regulated genes suggest that genes related to anti-inflammatory response and anti-apoptotic response were activated including *STAT1*, *IRAK1*, *ATK1*, *I*κBα, *CCNG2* and *Bcl*-2. Functional clustering results of down-regulated genes suggest that chromatin remodeling associated processes were inhibited. Notably, hematopoietic processes, especially B cell activation or possibly lymphoid lineage, were inhibited due to down-regulation of cell adhesion. A selected subset of DLEU1 target genes with biological function were validated by real time qRT-PCR. The results indicated a pattern consistent with gene expression profiling data (Figure 5D).

### Effect of DLEU1 on sensitivity of xenograft tumors to RTX and/or CTX *in vivo*

**Figure 6:**
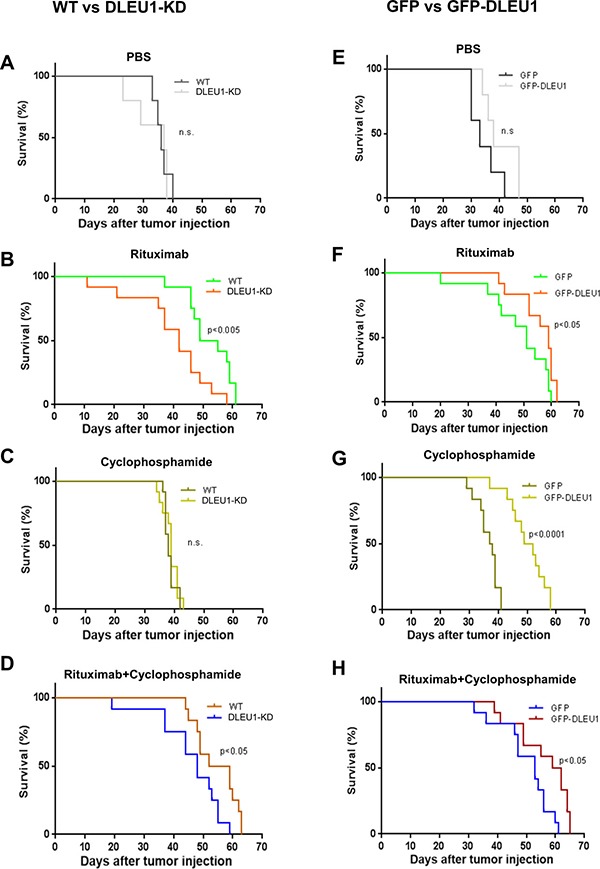
Survival of DLEU1-KD and GFP-DLEU1 mice treated with rituximab, cyclophosphamide and rituximab/cyclophosphamide combination (**A**–**D**) Pair-wise comparison of survival curves of WT and DLEU1-KD mice (A–D) following the treatment of RTX, CTX, or RTX/CTX. *p < 0.05; **p < 0.005. (**E**–**H**) Pair-wise comparison of control (GFP) and DLEU1 overexpressed (GFP-DLEU1) xenograft mice (E–H) following the treatment of RTX, CTX, or RTX/CTX. ns, not significant; *p < 0.05; **p < 0.005; ***p < 0.0001.

We further investigated whether *DLEU1* expression level has any effect on the sensitivity to RTX and/or CTX treatment of xenografted NSG mice. DLEU1-KD or WT Raji cells engineered to express luciferase were injected into NSG mice. Xenografted mice were then treated with RTX (30 mg/kg), CTX (25 mg/kg), or RTX together with CTX, weekly for 4 weeks. Mice receiving PBS served as a vehicle control. We found that tumors formed by DLEU1-KD cells grew faster than those by WT cells under the treatment of RTX, CTX, or combined (Supplementary Figure 2), indicating that the DLEU1 knockdown cells were more resistant to the treatment of these drugs. Consistently, we found that DLEU1-KD cells injected mice had significantly shortened survival time compared to those injected with WT cells when treated with RTX (42 days vs. 52 days, *p* < 0.005), or RTX combined with CTX (48 days *vs* 55.5 days, *p* < 0.05) (Figure 6A–6D). In the GFP-DLEU1 overexpressed xenograft model, we found that GFP-DLEU1 mice had significantly extended survival compared to GFP mice following treatment of RTX (57.5 days *vs* 51 days, *p* < 0.05, *n* = 8 per group), CTX (50.5 days *vs* 37.5 days, *p* < 0.0001, *n* = 12 per group), and RTX/CTX-combination treatment (60.5 days *vs* 53 days, *p* < 0.05, *n* = 12 per group) (Figure 6E–6H and Supplementary Figure 3).

## DISCUSSION

Children and adolescents with 13q14.3 deletion have a significant poorer EFS compared to pediatric BL patients without this abnormality [10, 11]. Furthermore, this region has been determined to be the most frequently genetically altered locus in B-CLL [24, 25] and a major control locus of B-cell proliferation in CLL [24]. In the current preclinical study, we investigated if DLEU1 expression alters 1) BL programmed cell death, cell proliferation and the expression of DLEU1 network genes *in vitro* and 2) the survival of DLEU1 knockdown and overexpressing BL xenografted mice following chemoimmunotherapy.

We generated TALENs-mediated DLEU1 KD (83% reduction, *p* < 0.01) as well as GFP-DLEU1 stable expressing Raji BL cell lines. We investigated the effect of DLEU1 expression on cell proliferation and programmed cell death, and analyzed the expression of network genes and signaling pathways in BL following treatment with RTX and/or CTX using qRT-PCR and western blotting analyses. Gene expression profiling was performed using Affymetrix microarrays in DLEU1 KD BL cells compared to control to identify cellular processes regulated by DLEU1. We found that DLEU1 in part regulates BL apoptosis, cell proliferation and associated signaling pathways. Gene expression data additionally support the hypothesis that DLEU1 plays in part a role in BL cell signaling by demonstrating that anti-inflammatory and anti-apoptotic response genes, *STAT1*, *IRAK1*, *ATK1*, *IκBα*, *CCNG2* and *Bcl-2* were activated whereas hematopoietic-associated and chromatin remodeling-associated genes were downregulated in DLEU1-KD BL in comparison to wild type BL cells. These observations are consistent with the recent report of tumor suppressive function within the genetic region of 13q14.3 which is associated with suppressing tumor cell viability in cancers [24].

Furthermore, we observed a significant decrease in both chemotherapeutic and immunotherapeutic responses of DLEU1 KD cells as compared to WT with CTX and RTX, respectively, suggests that a DLEU1 loss may in part be a potential mechanism for therapeutic resistance. We then established *in vivo* xenograft NSG mouse models with DLEU1 knockdown and overexpressing Raji cells and investigated the sensitivity of these xenograft tumors to the treatment of RTX and/or CTX. More importantly, the deletion of *DLEU1* results in significant enhancement of tumor progression and shortened survival in RTX and CTX treated DLEU1 KD cells xenografted mice compared to WT cell injected mice. In contrast, GFP-DLEU1 BL xenografted mice treated with RTX and/or CTX exhibited a significant survival advantage over control BL xenografted mice. These data in part suggest that DLEU1 level is associated with chemoimmunotherapy resistance.

The use of a TALENs method provided an opportunity to investigate the loss of function of a specific gene [26–29]. While this study sheds some light on at least one mechanism of chemoimmunotherapy resistance in BL, it will be important to investigate the role of other gene products found in the 13q14.3 region such as microRNA clusters, *miR-15a/miR-16-1* [24, 30], and long non-coding RNA (lncRNA) gene, *DLEU2* [24], and *RFP2* and/or *KCNRG* [31]. *DLEU1* may perhaps work in tandem with *miR-15a/miR-16-1*, *DLEU2* and/or *RFP2* to create an additive or synergistic tumor suppressive or oncogenic effect. We plan to investigate in future studies the individual and combined roles of *miR-15a*, *miR-16-1, DLEU2* and/or *RFP2* by molecular excision via TALENs in BL lymphomagenesis and their importance in regulating BL programmed cell death.

Interestingly, *DLEU1* has been considered as a lncRNA gene [16, 17, 24]. However, *DLEU1* was defined as putative functional protein coding gene that encodes a 78 amino acids based on Ensemle database [32] and the universal protein resource (UniProt) [33, 34]. It has recently been implicated that predicted open reading frame in lncRNA can be translated to functional peptides [35, 36]. A human protein-protein interaction study showed an interaction between DLEU1 and TUBB2C, TUBB2C and Bruton’s tyrosine kinase (BTK) [37, 38], suggesting that DLEU1 may interact with BTK. BTK is a regulator of normal B-cell development and is activated upon B-cell receptor (BCR) stimulation [39]. In addition, BTK has recently become an important target in hematological malignancies, with the development of various targeted therapies specifically targeted to inhibit BCR signaling [40, 41]. Specifically, a BTK-targeting molecule, ibrutinib has been approved by FDA for treatment of CLL and MCL patients who have failed prior therapy and is currently under pre-clinical investigation for treatment of BL [42]. We aim to further investigate the potential connection between DLEU1 and the BCR signaling pathways, and the role of this relationship in BL.

In summary, our results suggest that the deletion of DLEU1 at 13q14.3 in BL may in part result in chemoimmunotherapy resistance in BL. Furthermore, there is also the implication for pretreatment screening for the presence of the 13q14.3 deletion in children and adolescent BL and the potential investigation of alternative therapeutic strategies. In future studies, we will address which therapies may perhaps re-sensitize BL cells lacking the 13q14.3 region to improve therapy induced programmed cell death.

## MATERIALS AND METHODS

### Ethics statement

Investigation has been conducted in accordance with the ethical standards and according to the Declaration of Helsinki and according to national and international guidelines and has been approved by the Institutional Animal Care and Use Committee at New York Medical College.

### Engineering of transcription activator-like effector nucleases (TALENs) targeting DLEU1

Potential TALENs target sites of *DLEU1* were identified using web-based ZiFiT Targeter software (http://zifit.partners.org/). The BLAST analysis and Repeat Masker Web server (www.repeatmasker.org) were utilized for uniqueness of targeted sequences. The designed *DLEU1* TALEN pairs were constructed based on Restriction Enzyme And Ligation (REAL) assembly methods using the Joung Lab REAL Assembly TALENS kit from Addgene [43]. TALENs were designed to target the *DLEU1* transcription start and stop site on chromosome 13q14.3. To examine the validity of constructed TALENs pairs in mammalian cells, plasmids expressing each TALENs pair were transfected into Human embryonic kidney (HEK)293 cells and genomic DNA was extracted 24 hours post-transfection. Disrupted target sequences caused by non-homologous end joining (NHEJ) were confirmed by surveyor mutation detection assay (Supplementary Figure 1) and sequencing analysis (data not shown).

### Cell culture and transfection

HEK293 and Raji human BL cells were obtained from ATCC (Manassas, VA) and maintained as previously described [44, 45]. These cell lines were authenticated by ATCC, and were provided us within 6 months before the use of cells, and cells were passaged for fewer than 8 weeks of resuscitation in our laboratory. Plasmid constructs were transfected into HEK293 and Raji cells using Lipofectamine^®^ reagent (Invitrogen) and Amaxa Nucleofector^™^ Kit V (Lonza), respectively according to the manufacturer instructions.

### Surveyor nuclease assay

Genomic DNA was extracted from cells 24–48 hours post-transfection using a genomic DNA extraction kit (Promega). The measurement of NHEJ - mediated mutation was performed using the Surveyor endonuclease assay kit (Transgenomic, Inc.) according to the manufacturer’s instructions. The PCR primers used are as follows: F2, 5′-TCTTGCTTTCCCGACATTT TTACG-3′, F4, 5′-CTAGAAGAGCCAACCAACAG-3′, and R1, 5′-AGTTGTTCCGAGGCTTAAGTGC GA-3′.

### Generation of TALENs mediated *DLEU1* knockdown cell line

Cells were transfected with TALEN expression plasmids and genomic DNA was extracted at 48 hours post-transfection followed by Surveyor nuclease assay as above. Raji cells with *DLEU1* gene modification identified by NHEJ were seeded into 96 well plates to isolate a single clone. Individual colonies were picked, expanded and re-genotyped as described above.

### Establishment of BL cell line stably overexpressing DLEU1

The DLEU1 overexpression construct (GFP-DLEU1) was generated by cloning the full length DLEU1 cDNA into pEGFP-N3 vector. GFP-DLEU1 was then transfected into 293T or Raji cells. Cells were selected under G418 (500 ug/ml) for stable clones and expression of GFP-DLEU1 was confirmed by qRT-PCR and Western blotting analyses.

### Quantitative reverse-transcriptase PCR (qRT-PCR)

Total RNA was prepared using TRIzol reagent (Invitrogen) according to the manufacturer’s instruction and 1 ug of RNA was used for cDNA synthesis using qScript^™^ cDNA Synthesis Kit (Quantas). qRT-PCR was performed on the CFX96 Real-time system (Bio-rad) using SsoFast^™^ EvaGreen^®^ Supermix (Bio-rad). Primers used in qRT-PCR are provided in Supplementary Table 2. Relative quantification (ddCt) of mRNA expression of genes was determined by normalizing to the housekeeping gene, glyceraldehyde-3-phosphate dehydrogenase (GAPDH).

### Western blotting analysis

Western blot analysis was performed as we have previously described [44]. Band intensities on SDS-PAGE gel were measured using ImageJ software program. Antibodies specific for GAPDH, phospho-Akt and Akt, phospho-IκBα and IκBα and GFP were purchased from Cell Signaling Technology and an antibody specific for DLEU1 was provided by ProteinTech. Antibodies specific for anti-apoptotic genes, Bcl-2, Bcl-xL, and Mcl-1 and pro-apoptotic proteins, Bad and Bax were purchased from Cell Signaling Technology.

### Cell proliferation assay

Prior to cell proliferation measurements, cells were treated without or with various doses of RTX (0 – 100 ug/ml), CTX (0 – 10 mM) alone and in combination. Cells (1 × 10^4^) were plated into 48 well plates and then counted every 24 hrs as previously described [44]. Cell growth was determined using the non-radioactive CellTiter 96^®^ Aqueous One solution cell proliferation assay (MTS) (Promega) and measured by a Multiabel Counter (Perkin Elmer) at OD490.

### Caspase 3/7 assay

Caspase 3/7 activity was directly measured at 48 and 72 hours after treatment using Caspase-Glo 3/7 Activity kit (Promega) according to the manufacturer’s protocol. Relative light intensity was measured in each well using Clarity Luminescence Microplate Reader (BioTek).

### Antibodies and reagents

A chimeric human and mouse anti-CD20 type I mAb, Rituximab (RTX), was purchased from Genentech Inc. and CTX was purchased from Sigma Aldrich and dissolved as a stock solution in PBS.

### Gene expression profiling

Gene expression profiling experiments were performed using Affymetrix Human Genome 133 Plus 2.0 arrays at the Rockefeller University Genomics Facility. Two hundred nanograms of total RNA were biotin-labeled using Ambion MessageAmp^™^ Premier RNA Amplification Kit (Life Technologies). The fragmented aRNA was hybridized to the array as described in the Affymetrix Technical Analysis Manual (Affymetrix). Gene Chips were stained with streptavidin-phycoerythrin, and then scanned with the Affymetrix GeneChip Scanner 3000 7G. Data has been deposited in the NCBI’s Gene Expression Omnibus data base (accession number GSE65674).

### Microarray data processing and analysis

Signal intensity was processed using MAS5 algorithm. The Probe sets that were not tagged as present in any samples according to the presence-absence-call and control probe sets were discarded. Among 21,437 surviving probe sets, we identified probe sets whose signal ratio between test group average and control group average exceeded two-fold as DEG [46] and retained for further analyses. We used DAVID (version 6.7) to investigate the biological processes and pathways implicated by DEG [47]. Specifically, we employed the functional annotation clustering utility that clusters functionally related terms linked to DEG. We interrogated over-expressed and under-expressed probe sets separately.

### *In vivo* xenograft models

All experimental animals were γ- irradiated (2.5 Gy) 1 day before cell transplantation. DLEU1-KD and mock control (WT), or GFP-DLEU1 and mock control (GFP) were transfected with a firefly luciferase expression plasmid (*ffluc-zeo*, kindly provided by Laurence Cooper MD, PhD) into Raji cells followed by zeocin selection for stable clones. These selected cells (1 × 10^6^) were then subcutaneously injected into four- to six-week-old female NSG (NOD.Cg-Prkdc^scid^ *Il2rg*tm1Wjl/SzJ) mice from Jackson laboratory. After verification of tumor burden by bioluminescence imaging using the Xenogen IVIS-200 (Caliper Life Sciences), mice were treated with either PBS control (*n* = 5 per group), or RTX (30 mg/kg, *n* = 12 per group), or CTX (25 mg/kg, *n* = 12 per group) or RTX together with CTX (*n* = 12 per group) by intraperitoneal (i.p) injection at 7 day intervals as we have previously described [48]. Tumor progression was monitored at day 7 and once every week by bioluminescence imaging. All mice were housed and maintained under specific pathogen-free conditions under protocols (51-2-0913H and 59-2-1113H) approved by the Institutional Animal Care and Use Committee at New York Medical College.

### Statistical analysis

Significant differences between DLEU1-KD vs WT or GFP-DLEU1 vs GFP groups (RT-PCR, cell proliferation assay, Caspase 3/7 assay and band intensity by Western blot) were determined by using one-tailed paired Student’s *t*-test in Microsoft Excel and *p*-values less than 0.05 were considered significant. Error bars are described in the Figure legends as ± SD. Survival rates were analyzed by the Kaplan-Meier method and differences evaluated by log-rank test using the Prism Version 6.0 software.

## SUPPLEMENTARY MATERIALS FIGURES AND TABLES







## Abbreviations

BCR: B-cell receptor
BFM: Berlin-Frankfurt-Munster
BL: Burkitt lymphoma
BTK: Bruton’s tyrosine kinase
CLL: chronic lymphocytic leukemia
CTX: cyclophosphamide
DEG: differentially expressed genes
DLBCL: diffuse large B-cell lymphoma
DLEU1: Deleted in Lymphocytic Leukemia 1
DLEU1-KD: DLEU1 knockdown
EFS: event free survival
FAB: French American British
GAPDH: glyceraldehyde-3-phosphate dehydrogenase
HEK: Human embryonic kidney
lncRNA: long non-coding RNA
NHEJ: non-homologous end joining
NHL: non-Hodgkin lymphoma
OS: Overall survival
qRT-PCR: Quantitative reverse-transcriptase PCR
REAL: restriction enzyme and ligation
RTX: rituximab
TALEN: transcription activator-like effector nuclease
TUBB2C: Tubulin beta-2C chain
UBR1: ubiquitin-protein ligase
WT: wild-type.
