# Supplementary material for: The effects of DLEU1 gene expression in Burkitt lymphoma (BL): potential mechanism of chemoimmunotherapy resistance in BL
Source: Oncotarget. 2017 Feb 24;8(17):27839–53. doi: 10.18632/oncotarget.15711 (PMC5438612; doi:10.18632/oncotarget.15711)
Supplement: Supplementary file 1 [file oncotarget-08-27839-s001.pdf]

The effects of DLEU1 gene expression in Burkitt lymphoma (BL): potential mechanism of chemoimmunotherapy resistance in BL

Supplementary Materials

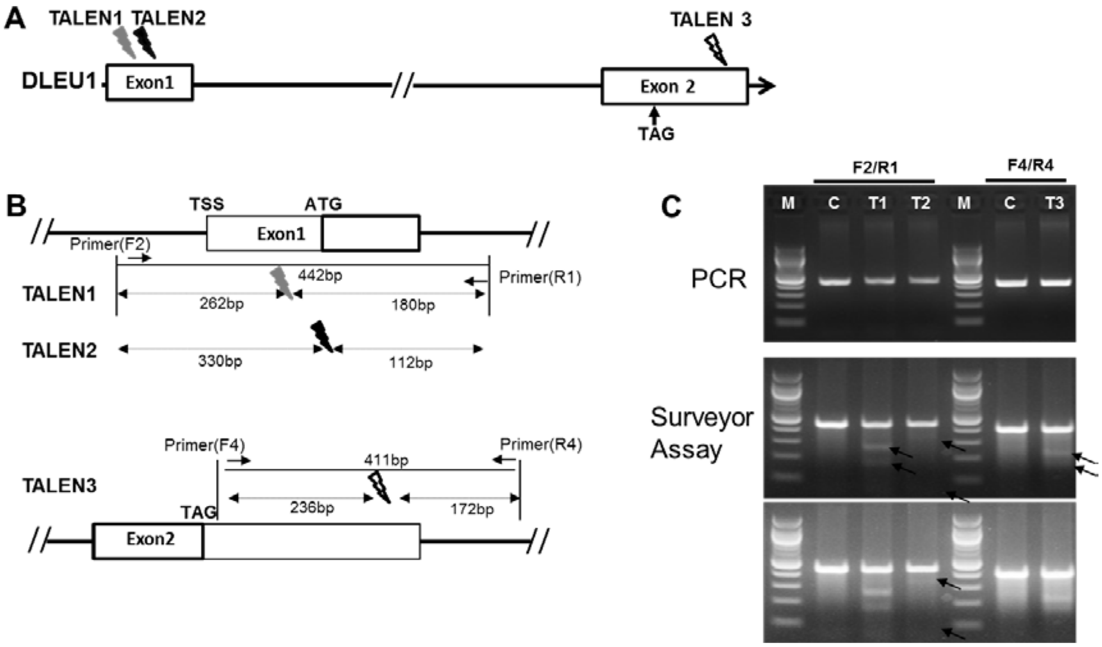

Supplementary Figure 1: Validation of DLEU1 TALENs by Surveyor nuclease assay in mammalian cells. PCR with primers spanning each of the TALEN targeting sites shows the expected 442 bp and 411 bp amplicon using primer F2/R1 and F4/R4, respectively (**A** and **B**). Each TALEN pair targeting DLEU1 was transfected into 293 cells and then collected at 24 hours post-transfection. DLEU1 locus encompassing each TALEN target site was amplified by PCR and the amplicons were then denatured and annealed to generate mismatch bubbles caused by non-homologous end joining (NHEJ) following performance of the surveyor nuclease assay (**C**) as described in Materials and Methods. *Cel*-I endonuclease was used to digest the 442 bp (TALEN1 and TALEN2) and 411 bp (TALEN3) heteroduplex DNA derived from pooled DNAs between WT and each targeted cells into 262 bp and 180 bp, 330 bp and 112 bp, and 236 bp and 172 bp by TALEN 1, TALEN 2 and TALEN 3, respectively (B). Green arrowheads on indicate the expected size of the fragmented PCR products and represent highly exposed gel picture (bottom) (C). M indicates 100 bp DNA ladder and C is WT.

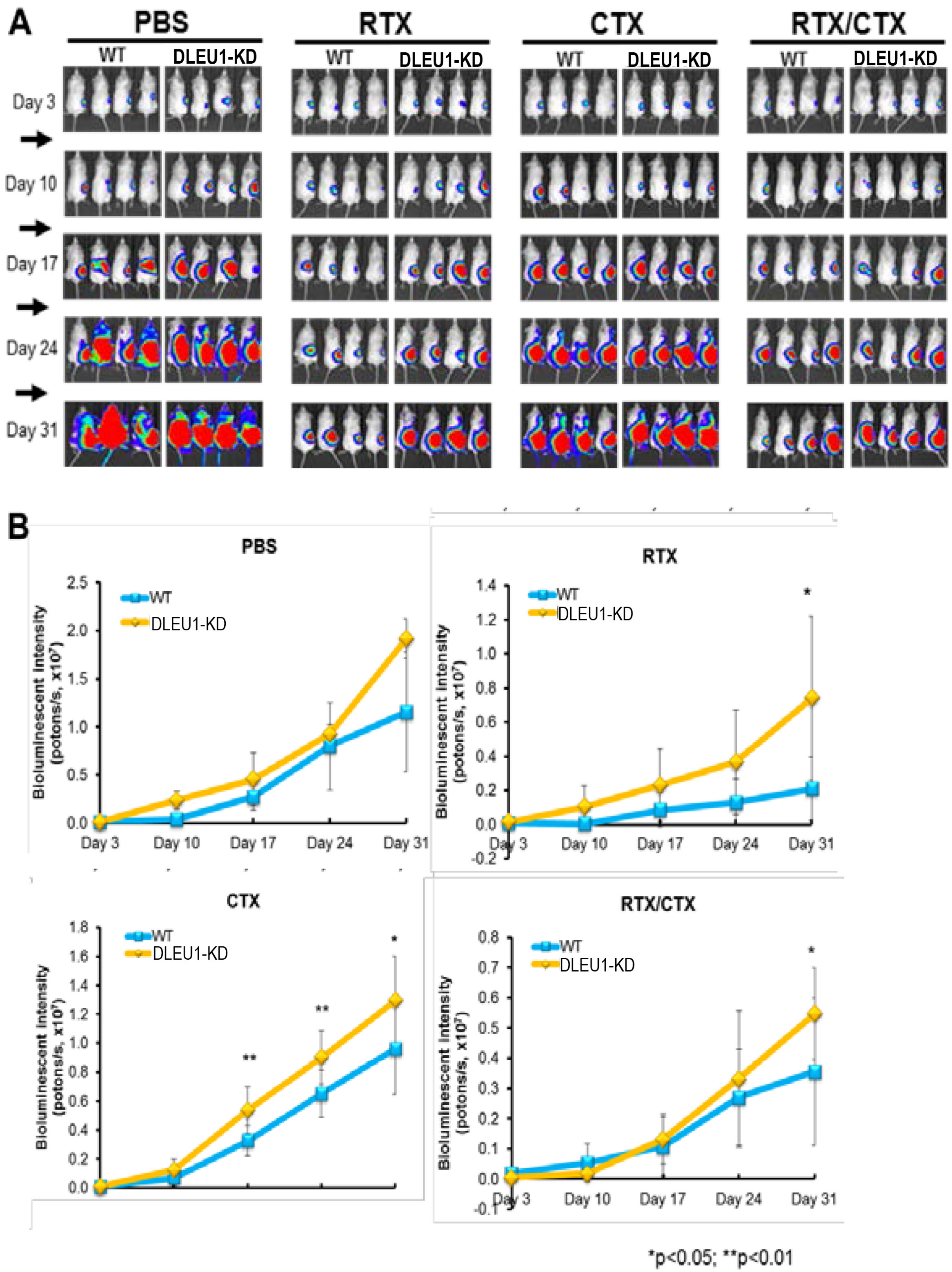

Supplementary Figure 2: Tumor progression from DLEU1-KD xenografted NSG mice. WT and DLEU1-KD Raji-Luc cells (1×10^6) were subcutaneously injected into NSG mice at day 0 (**A**). Three doses of rituximab (RTX), cyclophosphamide (CTX) and in combination RTX and CTX were injected by intraperitoneal (i.p) on days 4, 11 and 18, respectively. Mice treated with PBS served as a control. Representative bioluminescence images are shown. Photons emitted from luciferase-expressing cells were measured in regions of interest that encompassed the entire body and quantified using the Living Image software. Signal intensities (Bioluminescence) are shown at the time points indicated in PBS, RTX, CTX and RTX/CTX-treated DLEU1-KD mice (**B**). Error bars correspond to the mean ± SD. *P* values obtained with Student *t*-test, $*p < 0.01$; $**p < 0.05$.

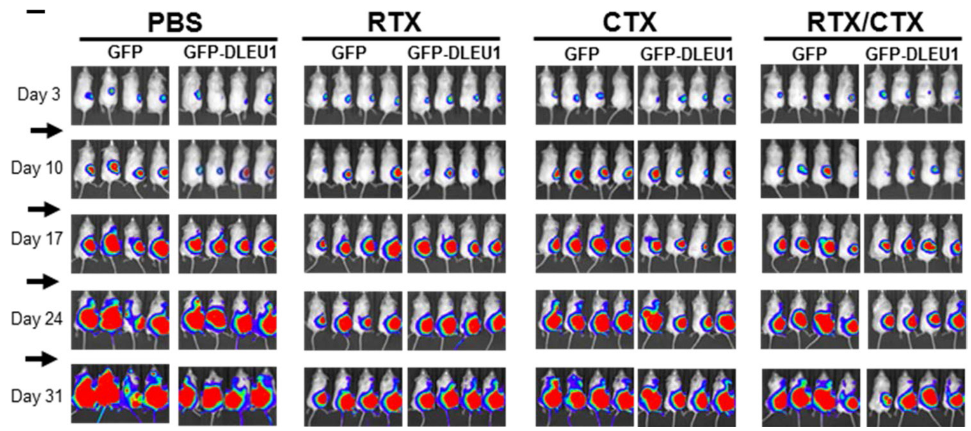

Supplementary Figure 3: Tumor progression of GFP and GFP-DLEU1 overexpression mice treated with rituximab (RTX), cyclophosphamide (CTX) and RTX/CTX combination (8 mice in each group). GFP and GFP-DLEU1 Raji-Luc cells (1×10^6) were subcutaneously injected into NSG mice at day 0. Three doses of RTX, CTX and in combination RTX and CTX were injected by intraperitoneal (i.p) on days 4, 11 and 18, respectively. Mice treated with PBS served as a control. Representative bioluminescence images are shown. Photons emitted from luciferase-expressing cells were measured in regions of interest that encompassed the entire body and quantified using the Living Image software. Signal intensities (Bioluminescence) are shown at the time points indicated in PBS, RTX, CTX and RTX/CTX-treated GFP-DLEU1 mice.

Supplementary Table 1: Significantly enriched GO terms of up- (left) and down (right)-regulated genes (2 folds, $p < 0.05$) in DLEU1 knockdown Raji. See_Supplementary_Table_1

Supplementary Table S2: Primers for RT-PCR. See_Supplementary_Table_2
