# Supplementary material for: The effects of DLEU1 gene expression in Burkitt lymphoma (BL): potential mechanism of chemoimmunotherapy resistance in BL
Source: Oncotarget. 2017 Feb 24;8(17):27839–53. doi: 10.18632/oncotarget.15711 (PMC5438612; doi:10.18632/oncotarget.15711)
Supplement: Supplementary file 2 [file oncotarget-08-27839-s002.docx]

**Supplementary Tables**

**Table S1.** Significantly enriched GO terms of up- (left) and down (right)-regulated genes (2 folds, p<0.05) in DLEU1 knockdown Raji

|  | **Genes (n)** |  | **Genes (n)** |
| --- | --- | --- | --- |
| **BIOLOGICAL PROCESS** |  | **BIOLOGICAL PROCESS** |  |
| activation of adenylate cyclase activity by G-protein signaling pathway | 4 | cartilage development | 4 |
| amino sugar metabolic process | 7 | cell activation | 12 |
| anti-apoptosis | 32 | cellular macromolecular complex assembly | 17 |
| apoptosis | 75 | cellular macromolecular complex subunit organization | 17 |
| aromatic compound biosynthetic process | 6 | chromatin assembly | 12 |
| biological adhesion | 39 | chromatin assembly or disassembly | 15 |
| cAMP-mediated signaling | 8 | chromatin organization | 19 |
| cell adhesion | 39 | chromosome organization | 22 |
| cell death | 87 | DNA packaging | 12 |
| cell-cell junction organization | 7 | extracellular structure organization | 6 |
| cellular amino acid derivative biosynthetic process | 7 | hemopoiesis | 13 |
| coenzyme biosynthetic process | 17 | hemopoietic or lymphoid organ development | 14 |
| cofactor biosynthetic process | 21 | immune response | 17 |
| cyclic-nucleotide-mediated signaling | 8 | immune system development | 14 |
| death | 88 | intracellular signaling cascade | 33 |
| defense response | 40 | leukocyte activation | 11 |
| embryonic skeletal system development | 8 | leukocyte differentiation | 9 |
| embryonic skeletal system morphogenesis | 7 | lymphocyte activation | 11 |
| entry into cell of other organism during symbiotic interaction | 4 | lymphocyte differentiation | 9 |
| entry into host | 4 | negative regulation of biosynthetic process | 21 |
| entry into host cell | 4 | negative regulation of cell communication | 11 |
| entry into other organism during symbiotic interaction | 4 | negative regulation of cell size | 6 |
| entry of virus into host cell | 4 | negative regulation of cellular biosynthetic process | 21 |
| G-protein signaling, coupled to cAMP nucleotide second messenger | 8 | negative regulation of gene expression | 23 |
| G-protein signaling, coupled to cyclic nucleotide second messenger | 8 | negative regulation of kinase activity | 7 |
| immune response | 55 | negative regulation of macromolecule biosynthetic process | 21 |
| inflammatory response | 29 | negative regulation of macromolecule metabolic process | 27 |
| maintenance of location | 12 | negative regulation of nitrogen compound metabolic process | 19 |
| movement in environment of other organism during symbiotic interaction | 4 | negative regulation of nucleobase, nucleoside, nucleotide and nucleic acid metabolic process | 19 |
| movement in host environment | 4 | negative regulation of protein kinase activity | 7 |
| negative regulation of apoptosis | 48 | negative regulation of signal transduction | 10 |
| negative regulation of caspase activity | 6 | negative regulation of transcription | 19 |
| negative regulation of catalytic activity | 36 | negative regulation of transcription, DNA-dependent | 14 |
| negative regulation of cell death | 48 | negative regulation of transferase activity | 7 |
| negative regulation of cellular protein metabolic process | 31 | nucleosome assembly | 12 |
| negative regulation of immune system process | 11 | nucleosome organization | 12 |
| negative regulation of molecular function | 44 | phosphoinositide-mediated signaling | 5 |
| negative regulation of programmed cell death | 48 | positive regulation of cell proliferation | 14 |
| negative regulation of protein metabolic process | 32 | protein-DNA complex assembly | 12 |
| negative regulation of protein transport | 8 | regulation of nervous system development | 7 |
| negative regulation of response to external stimulus | 7 | regulation of Rab GTPase activity | 5 |
| negative regulation of secretion | 6 | regulation of Rab protein signal transduction | 5 |
| negative regulation of transport | 17 | regulation of RNA metabolic process | 53 |
| nitrogen compound biosynthetic process | 46 | regulation of transcription | 71 |
| nucleobase, nucleoside and nucleotide biosynthetic process | 28 | regulation of transcription from RNA polymerase II promoter | 23 |
| nucleobase, nucleoside, nucleotide and nucleic acid biosynthetic process | 28 | regulation of transcription, DNA-dependent | 53 |
| nucleoside monophosphate biosynthetic process | 10 | response to hypoxia | 7 |
| nucleotide biosynthetic process | 28 | response to oxygen levels | 7 |
| oxidation reduction | 70 | second-messenger-mediated signaling | 9 |
| peptide metabolic process | 11 |  |  |
| positive regulation of adenylate cyclase activity by G-protein signaling pathway | 4 |  |  |
| positive regulation of endocytosis | 7 |  |  |
| positive regulation of myeloid cell differentiation | 9 |  |  |
| positive regulation of myeloid leukocyte differentiation | 6 |  |  |
| programmed cell death | 78 |  |  |
| protein heterooligomerization | 11 |  |  |
| pteridine and derivative biosynthetic process | 5 |  |  |
| pteridine and derivative metabolic process | 6 |  |  |
| purine nucleotide biosynthetic process | 22 |  |  |
| regulation of adenylate cyclase activity involved in G-protein signaling | 4 |  |  |
| regulation of apoptosis | 93 |  |  |
| regulation of cell death | 93 |  |  |
| regulation of muscle contraction | 8 |  |  |
| regulation of myeloid cell differentiation | 14 |  |  |
| regulation of myeloid leukocyte differentiation | 8 |  |  |
| regulation of programmed cell death | 93 |  |  |
| response to lipopolysaccharide | 11 |  |  |
| response to wounding | 39 |  |  |
| ribonucleotide biosynthetic process | 21 |  |  |
| sequestering of metal ion | 4 |  |  |
| sulfur metabolic process | 20 |  |  |
|  |  |  |  |
| **CELLULAR COMPONENT** |  | **CELLULAR COMPONENT** |  |
| cytoplasmic membrane-bounded vesicle | 53 | chromatin | 13 |
| cytoplasmic vesicle part | 19 | chromosomal part | 18 |
| envelope | 92 | extracellular region | 24 |
| extracellular region | 75 | integral to membrane | 84 |
| extracellular region part | 37 | integral to plasma membrane | 20 |
| integral to membrane | 328 | intrinsic to membrane | 86 |
| intrinsic to Golgi membrane | 8 | intrinsic to plasma membrane | 20 |
| intrinsic to membrane | 338 | nucleosome | 10 |
| organelle envelope | 92 | plasma membrane | 59 |
| organelle outer membrane | 19 | protein-DNA complex | 10 |
| outer membrane | 19 | synapse | 9 |
| plasma membrane | 208 |  |  |
| proteasome complex | 16 |  |  |
|  |  |  |  |
| **MOLECULAR FUNCTION** |  | **MOLECULAR FUNCTION** |  |
| actin binding | 34 | cation binding | 95 |
| calcium ion binding | 58 | DNA binding | 71 |
| carboxylesterase activity | 13 | enzyme inhibitor activity | 9 |
| cytokine activity | 11 | glycosaminoglycan binding | 5 |
| double-stranded RNA binding | 9 | GTPase activator activity | 11 |
| heat shock protein binding | 16 | GTPase regulator activity | 20 |
| lipase activity | 11 | heparin binding | 5 |
| O-acyltransferase activity | 9 | ion binding | 96 |
| peptide binding | 17 | lipid binding | 14 |
| phospholipase activity | 8 | metal ion binding | 94 |
| phospholipid binding | 22 | nucleoside-triphosphatase regulator activity | 20 |
| proteasome regulator activity | 4 | pattern binding | 5 |
| protein kinase C binding | 8 | polysaccharide binding | 5 |
| protein tyrosine/serine/threonine phosphatase activity | 10 | Rab GTPase activator activity | 5 |
| sulfotransferase activity | 7 | Ras GTPase activator activity | 7 |
| transferase activity, transferring sulfur-containing groups | 9 | serine-type endopeptidase inhibitor activity | 4 |
| unfolded protein binding | 23 | transcription factor activity | 26 |
|  |  | transcription regulator activity | 45 |
|  |  | transition metal ion binding | 78 |
|  |  | zinc ion binding | 69 |
