# Supplementary material for: The effects of DLEU1 gene expression in Burkitt lymphoma (BL): potential mechanism of chemoimmunotherapy resistance in BL
Source: Oncotarget. 2017 Feb 24;8(17):27839–53. doi: 10.18632/oncotarget.15711 (PMC5438612; doi:10.18632/oncotarget.15711)
Supplement: Supplementary file 3 [file oncotarget-08-27839-s003.docx]

**Table S2.** Primers for RT-PCR

| **Gene** | **Name** | **5'-3'** | **Product size** | **Gene bank** |
| --- | --- | --- | --- | --- |
| DLEU1 | Forward | CGTGCATTTAAAACCGCC | 159bp | NR_002605 |
|  | Reverse | TGTCTGCATTGTGACTCAATTC |  |  |
| GAPDH | Forward | ATGATGACATCAAGAAGGTGGTG | 222bp | NM_002046.4 |
|  | Reverse | CCATGAGGTCCACCACCCTGTTG |  |  |
| c-myc | Forward | GGATTCTCTGCTCTCCTCGA | 170bp | NM_002467 |
|  | Reverse | ACCTTTTGCCAGGAGCCTGCC |  |  |
| DLEU2 | Forward | GGTCTAAGTACCAGTAGTTTAG | 165bp | NR_002612 |
|  | Reverse | CTCTGGCTTGCTAACTTAAGTG |  |  |
| RASSF1 | Forward | AAAATGACTCTGGGGAGGTGA | 149bp | NM_007182 |
|  | Reverse | CAGGGCCTCTTGGATCTTCTGGC |  |  |
| UBR1 | Forward | CTGGTTGAAGGTAAAGCCAGA | 138bp | NM_174916 |
|  | Reverse | CCAGACCAAATGGAGCTTCCG |  |  |
| TUBB2C | Forward | GGACAACTTCGTTTTCGGTCA | 151bp | NM_006088 |
|  | Reverse | GTGGGTCAGCTGGAAACCCTGC |  |  |
| JAK2 | Forward | TGAGTCAACCAGGCATAATGTACT | 93bp | NM_004972 |
|  | Reverse | ATTCCATGCCGATAGGCTCT |  |  |
| Bcl-2 | Forward | AGTACCTGAACCGGCACCT | 74bp | NM_000633 |
|  | Reverse | GCCGTACAGTTCCACAAAGG |  |  |
| Bcl-XL | Forward | CCCAGGGACAGCATATCAG | 104bp | NM_004972 |
|  | Reverse | AGCGGTTGAAGCGTTCCT |  |  |
| Mcl-1 | Forward | ATTATCTCTCGGTACCTTCGG | 171bp | NM_021960 |
|  | Reverse | GATGTCCAGTTTCCGAAGCATG |  |  |
| CD20 | Forward | TGAATGGGCTCTTCCACA | 152bp | NM_152866 |
|  | Reverse | GGAGTTTTTCTCCGTTGC |  |  |
| Pim-1 | Forward | CCTGCTGTATGATATGGTGT | 193bp | NM_002648 |
|  | Reverse | GAGAACATCTTGCATCCATG |  |  |
| SOCS1 | Forward | CCCCTGGTTGTTGTAGCAG | 62bp | NM003745 |
|  | Reverse | GTAGGAGGTGCGAGTTCAGG |  |  |
| SOCS3 | Forward | AGACTTCGATTCGGGACCA | 128bp | NM003955 |
|  | Reverse | AACTTGCTGTGGGTGACCA |  |  |
| Bax | Forward | CAAGACCAGGGTGGTTGG | 84bp | NM_138761 |
|  | Reverse | CACTCCCGCCACAAAGAT |  |  |
| Bad | Forward | cgagtttgtggactcctttaaga | 108bp | NM_004322 |
|  | Reverse | caccaggactggaagactcg |  |  |
| p53 | Forward | aggccttggaactcaaggat | 85bp | NM_000546.4 |
|  | Reverse | ccctttttggacttcaggtg |  |  |
| TCF3 | Forward | cgcagttcggaggttcag | 78bp | NM_003200.3 |
|  | Reverse | aggaggagctgctctggtc |  |  |
| ID3 | Forward | catctccaacgacaaaaggag | 116bp | NM_002167.4 |
|  | Reverse | cttccggcaggagaggtt |  |  |
| CCND3 | Forward | ggtcacctgacgaggaggta | 84bp | NM_001136017.2 |
|  | Reverse | ggtagcgatccaggtagttca |  |  |
| PTEN | Forward | gcacaagaggccctagatttc | 77bp | NM_000314.4 |
|  | Reverse | cgcctctgactgggaatagt |  |  |
| NcoR2 | Forward | gcgaggtctccctgagtctt | 95bp | NM_006312.5 |
|  | Reverse | ccagtcctcgtcatcagctc |  |  |
| STAT1 | Forward | tgagttgatttctgtgtctgaagtt | 92bp | NM_007315.3 |
|  | Reverse | acacctcgtcaaactcctcag |  |  |
| STAT3 | Forward | cccttggattgagagtcaaga | 106bp | NM_139276.2 |
|  | Reverse | aagcggctatactgctggtc |  |  |
| NFKBIA | Forward | gtcaaggagctgcaggagat | 110bp | NM_020529.2 |
|  | Reverse | atggccaagtgcaggaac |  |  |
| AKT1 | Forward | agcccacagagacagagacc | 78bp | NM_032375.3 |
|  | Reverse | cgtcctcatccatcacaaag |  |  |
| MDM2 | Forward | gactccaagcgcgaaaac | 63bp | NM_002392.3 |
|  | Reverse | cagacatgttggtattgcacatt |  |  |
| CCNG2 | Forward | gggggttgttttgatgaaagt | 91bp | NM_004354.2 |
|  | Reverse | gatcactgggaggagagctg |  |  |
| IRAK1 | Forward | gagaccttggctggtcagag | 110bp | NM_001569.3 |
|  | Reverse | gtgcttctcaaagccactcc |  |  |
